# Radiologic Outcomes of Intramedullary Nailing in Infraisthmal Femur-Shaft Fracture with or without Poller Screws

**DOI:** 10.1155/2019/9412379

**Published:** 2019-05-08

**Authors:** Sang-Heon Song

**Affiliations:** Department of Orthopaedic Surgery, Myongji Hospital, Hanyang University College of Medicine, Republic of Korea

## Abstract

**Background:**

Intramedullary nails have been the treatment of choice for acute femur-shaft fractures in adults. However, the infraisthmal location has a high risk of nonunion and is easy to malalign. This study evaluated radiologic outcomes of initial supportive use of poller screws in combination with antegrade femoral nailing in infraisthmal femur-shaft fracture.

**Methods:**

A total of 49 patients who had undergone antegrade nailing with or without supportive poller-screw insertion for infraisthmal femur-shaft fracture were included in this retrospective cohort study (23 patients with poller screws in group 1 versus 26 patients without poller screws in group 2). Patient demographics including sex, age, classification, mechanism of injury, operative time, poller-screw time, and radiologic outcomes, including union rate, time to union, and number of malunions, were evaluated.

**Results:**

Union rate in group 1 (95.6%) was significantly higher than that in group 2 (84.6%) (*p* = 0.04). Time to union was 19.8 weeks in group 1 and 20.3 weeks in group 2 (*p* = 0.31).

**Conclusion:**

Initial supportive insertion of two poller screws after nailing took a mean of 21minutes additionally but could lessen the risk of nonunion significantly in this study. We believe that these findings may have important clinical relevance for the treatment of infraisthmal femur-shaft fracture.

## 1. Introduction

Among the orthopedic high-energy injuries, femur-shaft fractures are common in clinical fields. According to the Swedish nationwide study, the annual incidence of femur-shaft fractures is 10 per 100,000 person-years [1]. Intramedullary nails have been the treatment of choice for acute femur-shaft fractures in adults [2]. However, femur-shaft fractures may have various characteristics that depend on the location of fracture, degree of bony comminution, and injured muscle envelopes. The reported risk of nonunion after nailing in femur-shaft fracture is 0.5%-12.5% [3–6]. A major risk factor for nonunion is fracture location. Park* et al.* reported that the location of the fracture was the only significant risk factor for the failure after exchange nailing in 41 cases of femoral nonunion. They concluded that additional procedures should be considered for nonisthmal fractures [7].

The poller blocking screw, which was devised by Kretteck* et al.*, could decrease the width of the medullary cavity and guide and block the nail in the center of the widened metadiaphyseal flaring area [8–12]. It could also increase the mechanical stiffness of the bone-implant construct in the tibia [13]. We have experienced favorable outcomes of antegrade femur nailing with additional poller screws for the initial treatment of infraisthmal fracture. The purpose of this study was to evaluate the radiologic outcomes after antegrade nailing with or without poller screws for infraisthmal femur-shaft fractures. We hypothesized that initial insertion of poller screws could have benefit for the union of acute infraisthmal femur-shaft fractures.

## 2. Materials and Methods

This was a single-center, retrospective cohort study conducted at the Department of Orthopaedic Surgery of a tertiary healthcare hospital. The study was approved by our institutional review board prior to its initiation, and informed consent was obtained from each patient prior to the study.

An electronic chart was reviewed retrospectively for the patients who had undergone antegrade femoral nailing for femur-shaft fracture between January 2009 and January 2017. Inclusion criteria were all infraisthmal acute femur-shaft fractures treated with antegrade nailing with or without poller blocking screws. Pathologic fractures, bisphosphonate-related atypical fractures, adolescent patients, deep intramedullary infection cases, and patients for whom there were insufficient available radiographs until union were excluded. Infraisthmal femur-shaft fracture was defined as a fracture located between just distal to isthmus portion and proximal to the supracondylar area.

A total of 49 patients met the inclusion criteria and were evaluated. The 23 patients who underwent nailing with poller screws were classified as group 1, and the 26 patients who underwent nailing without poller screws were classified as group 2. Patient demographics were recorded, including age, sex, classification (AO/OTA), mechanism of injury, open or closed fracture, other associated injuries in case of polytrauma, operative time from skin to skin, and time for the additional poller-screw insertion.

**Figure 1:**
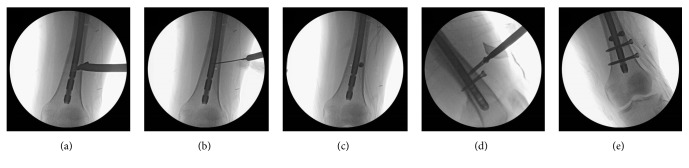
Intraoperative C-arm images show the procedure and position of poller-screw insertion. (a) Find the location of poller screws medial and lateral to the intramedullary nail in the metaphyseal area and drill a hole. (b) Confirm the position of the drill hole. (c) Insert one poller screw and make another drill hole. (d) Insert poller screws under C-arm guidance. (e) Confirm the final position after insertion of interlocking screws.

All patients were operated on by the author (Sang-Heon Song). A lateral starting antegrade titanium nail (TRIGEN TAN nail; Smith & Nephew, Memphis, TN, USA) was used, using a fracture table with supine position for all patients. All fractures were reduced closely by use of a joystick instrument. In group 1, two 5.0 mm cortical screws were used as poller screw anteroposteriorly in the metadiaphyseal flaring area, 2 or 3cm above the distal interlocking screw holes (Figure 1).

Postoperatively, a non-weight-bearing crutch gait was started on day 3 with continuous passive motion exercise of hip and knee. Patients were followed up at 1, 2, 4, 6, 9, and 12 months and at intervals of 6 months thereafter. Timing of partial-to-full weight-bearing was decided according to the follow-up radiographs.

Outcome measurements included were (1) union rate, (2) time to union, and (3) number of malunions. Union was defined as the ability to bear full weight without pain, with callus bridging in 3 of 4 cortices on radiographs. Malunion was defined as more than 5 degrees of angulation on radiographs, more than 15 degrees of malrotation compared to contralateral side range of motion, and shortening of more than 2 cm on scanographs.

Chi-square or Fisher's exact tests were used for categorical variables, and two-sample* t*-tests, ANOVA, and Wilcoxon rank-sum tests were used for continuous variables between the two groups. All statistical tests were performed using the SPSS for Windows (release 12.0) (SPSS, Inc., Chicago, IL, USA), and* p*<0.05 was considered significant.

## 3. Results

There were 30 male and 19 female patients. The mean age of all patients was 39.6 ± 12.1 (SD) years. Mean follow-up was 17.8 ±2.1 (SD) months. Initial fracture classification was described in Table 1. All fractures were 32 (AO/OTA classification: femur-diaphysis) and the distributions of A/B/C were described in Table 1. There were no statistically significant differences between the two groups for any parameter.

The mean operating time from skin incision to wound closure was 98.3 ± 21.2 (SD) min for group 1 (including poller time of 21.3 ± 6.1 min) and 76.8 ± 20.4 min for group 2, which had significant differences (*p* = 0.03). Five patients (one in group 1 and four in group 2) had failure to unite. Union rate was 95.7% in group 1 and 84.6% in group 2, which had statistically significant differences (*p* = 0.04). Time to union was 19.8 ± 3.2 (SD) weeks in group 1 and 20.3 ± 3.0 (SD) weeks in group 2, but there was no significant difference (*p* = 0.31).

Of the four patients with nonunion in group 2, three were hypertrophic nonunion and underwent additional poller screw insertion, and union was obtained without any further procedures. The other nonunion patient in group 2 had atrophic nonunion, which was a type B fracture, and initially underwent exchange nailing, and union was obtained 22 weeks later. The nonunion patient in group 1 had oligotrophic nonunion, with distal interlocking screw breakage three weeks after the initial surgery. Distal interlocking screws were reinserted and two additional poller screws were inserted simultaneously; union was achieved 18 weeks later.

The number of malunions was 6 (two patients in group 1 and four patients in group 2). Among the four cases in group 2, three patients had 5 to 7 degrees of varus angulation, and one had 17 degrees of external rotational deformity due to initial insufficient reduction. The two malunion cases in group 1 were less than 20 degrees of malrotation due to initial insufficient reduction. However, no case of malunion needed further surgical procedures, because they had no clinical discomfort in their daily lives (Table 2).

Two patients had surgical site infection (one in each group). Both of them had a gluteal bursal-site infection at the entry point; so incisional drainage and irrigation followed by intravenous antibiotics were done, which resolved the problem. All had full range of motion of hip and knee joints at the time of the last follow-up. None had any problems of quadriceps muscles at the site of the poller-screw insertion in group 1.

## 4. Discussion

Most femur-shaft fractures can be treated with intramedullary nailing successfully. However, popularities and a widened indication of femoral nailing in more complicated high-energy injuries have increased the rates of nonunion and delayed union. The reported risk of nonunion after nailing in femur-shaft fractures is 0.5%-12.5% [3, 4]. Most anatomical locations of nonunion are lower than the isthmic portion, because in this area the expanding diaphysis going to the metaphysis increases the size of the intramedullary canal. The usual antegrade nailing might not provide enough stability for healing in this infraisthmal area. Angular deformities due to malreduction and malunion could happen easily in this area.

The poller blocking screw, which was introduced by Kretteck* et al. *[8–12], was thought to work by narrowing the medullary canal in the metaphysis to provide a tight mechanical fit for the intramedullary nail. Stedtfeld* et al.* demonstrated the logic and clinical applications of blocking screws in 48 patients with long-bone fracture with various illustrations. They concluded that the use of transmedullary blocking screws in combination with an intramedullary nail is an important additional option that can be used to improve reduction and fixation of long-bone fractures at the metadiaphyseal junction. They commented that the blocking screw around the nail relieves axial strain in the fixation construct, and the interlocking screws through the nail control length and rotation [14]. In this study, all the fractures were located in the infraisthmal area of the femur, which is the transitional flaring zone from diaphysis to metaphysis, and initial insertion of two poller screws in combination with an intramedullary nail acted the same way as in the previously reported references.

In this study, we demonstrated the effect of poller screws in combination with an antegrade intramedullary nail in infraisthmal femur-shaft fractures. Initial supportive insertion of two poller screws after nailing took a mean of 21 minutes additionally but could lessen the risk of nonunion significantly. We believe that these findings may have important clinical relevance for the treatment of infraisthmal femur-shaft fractures. In addition, the time to union was about a week shorter in the poller group than in the nailing-only group even though there was no statistically significant difference.

The main limitation of this study was that there were few enrolled patients, not enough to do a valid multivariate analysis. Also, we applied clinical measurement rather than torsional CT scans for the rotational malignment due to economic burden for the patients. Some errors could be hidden in the results of rotational malalignment for this study. Most of the fractures in this study could be also treated with retrograde nailing with or without additional poller screws. Antegrade nailing with or without poller screws was selected rather than retrograde nailing as surgeon's preference in this study. Various clinical and radiological results might be investigated in next study between two different types of nail selections. Another limitation was that all the nails used in this study had only two holes for the distal interlocking screw. Other nails that have three or four multiholes (like coronal, sagittal, or oblique holes) for interlocking screws are now commercially available. Various construct models, including nailing with two distal interlocking screws only, nailing with three or more screws, and nailing with additional poller screws, should be compared in terms of the quality of reduction, union rate, or mechanical stability in the next study. Ideal guidelines could be developed for antegrade intramedullary nailing for the treatment of infraisthmal femur-shaft fractures with supportive additional poller screws.

## Figures and Tables

**Table 1:** Patient demographics*∗*.

	Overall (n = 49)	Group 1 (n = 23)	Group 2 (n = 26)
Sex			
Male	30	14	16
Female	19	9	10
Age, mean# (years)	39.6 ± 12.1	39.2 ± 11.8	39.8 ± 12.5
Classification			
32A	18	8	10
32B	21	10	11
32C	10	5	5
Open fracture	9	5	4
Follow-up, mean# (months)	17.8 ± 2.1	18.3 ± 2.0	17.5 ± 2.1

*∗*The differences between the groups were not statistically significant for all parameters. #The values are given as the mean and the standard deviation.

**Table 2:** Clinicoradiological results showing union rate, operative time including poller time, time to union, number of malunions, and range of motion.

Overall	Op. time*∗* (min)	Poller time*∗* (min)	Nonunion (n)	Time to union*∗* (wk)	Malunion (n)
Group 1	98.3 ± 21.2	21.3 ± 6.1	1	19.8 ± 3.2	2
Group 2	76.8 ± 20.4	-	4	20.3 ± 3.0	4

p value	*0.03*	-	*0.04*	0.31	0.23

*∗*The values are given as the mean and the standard deviation.

## Data Availability

The radiographic data used to support the findings of this study are available from the corresponding author upon request.
